# System‐failing creativity in health care

**DOI:** 10.1002/lrh2.10437

**Published:** 2024-06-24

**Authors:** Stijn Horck, Rachel E. Gifford, Bram P. I. Fleuren, Cheryl Rathert, Tracy H. Porter, Afshan Rauf, Yuna S. H. Lee

**Affiliations:** ^1^ Research Centre for the Education and Labour Market Maastricht University Maastricht The Netherlands; ^2^ Department of Health Services Research, Faculty of Health, Medicine and Life Sciences Maastricht University Maastricht The Netherlands; ^3^ Department of Work and Social Psychology, Faculty of Psychology and Neuroscience Maastricht University Maastricht The Netherlands; ^4^ Department of Health Management and Policy, College for Public Health and Social Justice Saint Louis University St. Louis Missouri USA; ^5^ Department of Management, Monte Ahuja College of Business Cleveland State University Cleveland Ohio USA; ^6^ Faculty of Business and Law University of Wollongong Wollongong New South Wales Australia; ^7^ Department of Health Policy and Management, Mailman School of Public Health Columbia University New York New York USA

**Keywords:** creativity, failure, health care management, well‐being

## Abstract

**Introduction:**

Health care professionals often generate novel solutions to solve problems during day‐to‐day patient care. However, less is known about generating novel and useful (i.e., creative) ideas in the face of health care system failure. System failures are high‐impact and increasingly frequent events in health care organizations, and front‐line professionals may have uniquely valuable expertise to address such occurrences.

**Methods:**

Our interdisciplinary team, blending expertise in health care management, economics, psychology, and clinical practice, reviewed the literature on creativity and system failures in health care to generate a conceptual model that describes this process. Drawing on appraisal theory, we iteratively refined the model by integrating various theories with key concepts of system failures, creativity, and health care worker's well‐being.

**Results:**

The SFC model provides a conceptualization of creativity from front‐line care professionals as it emerges in situations of failure or crisis. It describes the pathways by which professionals respond proactively to a systems failure with creative ideas to effectively address the situation and affect these workers' well‐being.

**Conclusions:**

Our conceptual model guides health care managers and leaders to use managerial practices to shape their systems and support creativity, especially when facing system failures. It introduces a framework for examining system‐failing creativity (SFC) and general creativity, aiming to improve health care quality, health care workers' well‐being, and organizational outcomes.

## 1 INTRODUCTION

Creativity, essential for organizational innovation, is significant in numerous sectors, including health care.^1^ The COVID‐19 pandemic underscored the value of creativity when established protocols and procedures were challenged. For instance, health care professionals creatively adapted to integrating personal protective equipment into their routines to prevent infection transmission,^2^ which included innovative measures like pre‐packed protective gear and a “runner” system for rapid delivery.^3^ During these unprecedented times, the front‐line employees' potential was highlighted in generating solutions,^4^ underlining the necessity of creative responses when the system fails to perform as intended. Although health care workers have historically demonstrated their capacity for creative problem‐solving,^5^, ^6^ conceptual examinations remain relatively limited in health care, partly due to this sector's emphasis on standardization and risk reduction.^7^, ^8^ Specifically, health care's relatively institutionalized nature, marked by adherence to rules and protocols and bureaucratic and hierarchical structures, fosters a culture where risks are minimized and deviations discouraged. This inherently risk‐averse environment might have limited conceptual research into creative problem‐solving in health care.^9^ The limited studies in this domain are mostly conducted outside of the health care sector. Examples include innovation tournaments^10^ or Lean approaches to idea generation such as Kaizen boards.^11^ However, these strategies promote a form of creativity and overlook spontaneous creative responses to system failures, which often occur in health care.

### 1.1 Research interests

In the post‐COVID landscape, global health systems have been thrust into a period of rapid transformation and improvement. The extreme pressures exerted on these systems during the pandemic have highlighted their vulnerabilities, revealing that even well‐established systems might not be equipped to handle unforeseen challenges. Therefore, we focus on the following research question: How can we effectively harness and support creativity spurred by system failures to enhance global health systems and improve individual well‐being? Recognizing and fostering this type of creativity could be an essential strategy for enhancing organizational outcomes^12^, ^13^ and bolstering well‐being among health care workers, especially considering the growing prevalence of burnout among health care workers.^14^

## 2 METHODS

At the Academy of Management Annual Meeting 2021, our group met during a paper development workshop focused on developing international collaboration.^15^ A brainstorming session was initiated that identified crisis leadership and health care worker well‐being as key topics. During this session, the role of creativity was identified as a potentially significant explanatory factor in these discussions. Subsequent meetings in 2 months after this session included gathering articles on crisis leadership, well‐being, failure, and creativity. This was achieved through the personal networks, checking references‐in‐references, and purposefully searching with search strings based on these key terms in health care management, psychology, and creativity literature. The lack of a conceptual framework for understanding creativity in challenging circumstances in the health care setting was identified through the application of thematic coding to categorize and analyze the gathered literature, combined with frequent meetings. Over 2 years, we assessed various theories, models, and frameworks to explore connections between creativity, failure, individual well‐being, and the organizational elements of a health care organization (HCO). Throughout our research, we continually refined our model by leveraging the interdisciplinarity of the research team. By employing this iterative approach, we were able to integrate appraisal theory with a range of other theories, culminating in the development of a novel conceptual model. Please see Data S1 for a more detailed description of the methodological process.

## 3 SYSTEM‐FAILING CREATIVITY

We build on Amabile's^16^ definition of creativity—the generation of novel and useful ideas—to define system‐failing creativity (SFC) as *the generation of novel and useful ideas and approaches in response to a system failure*. For this paper, we focus on the localized systems of HCOs, such as hospitals. We describe these local systems as interrelated and interdependent elements or components within an HCO, aimed to organize the health care delivery processes. Although SFC is a new theoretical concept, there are numerous examples where individuals tap into this form of creativity at the HCO level. For example, the COVID‐19 pandemic saw critical care doctors and nurses creatively repurpose split‐respirators, baby monitors, snorkels, and hairdryer hoods for COVID‐19 care when medical supply chains were hampered. Primary care physicians were compelled to develop and test ideas to reduce their “in‐basket” after years of struggling to respond to an overwhelming volume of system notifications and patient medical advice requests that were contributing to their burnout.^4^

Our definition of a system failure is derived from integrating the conceptualization of error and failure of Reason^17^ as the “failure of planned actions to achieve a desired goal.” In this context, we define a system failure as *an event or situation where the HCO's established system, designed for efficient and high‐quality health care delivery, and failed to perform as intended*. SFC might appear similar to other forms of creativity, like responsive creativity—a response to specific situational needs and closed problems.^18^ It distinguishes itself from other forms of creativity by its context‐dependent nature, which presents an unplanned, open problem that must be addressed. For example, consider the scenario of a hospital that implemented a series of structural changes to improve the quality of care delivery. These included a lengthier triage system, a double verification for drug administrations, an increase in detailed documentation, and frequent feedback meetings. While each change was designed to improve the quality of health care delivery, the unforeseen cumulative effect created bottlenecks and overwhelmed the clinical staff, dramatically increasing ER waiting times. Eventually, this led to patients facing hours of delay. During this escalating scenario, it is evident that the significant increase in waiting time represents a failure from the HCO's viewpoint. However, it remains unclear at which point a health care worker may be triggered to think of creative solutions to address the situation.

Emergence of SFC relies on health care workers recognizing a system failure; therefore, individual health care workers act as “agents” of organizational awareness. However, it is unclear how individuals appraise the failures they encounter and the potential effects of this process in terms of individual behavior. We apply the sequential stages outlined in Lazarus and Folkman's appraisal theory,^19^ a foundational psychological theory of stress, to articulate how a system failure either sparks creativity or leads to inactivity and withdrawal behavior of health care workers within the HCO. Appraisal theory explains how individuals evaluate events through two stages: primary and secondary appraisals. Primary appraisals assess the events based on relevance and urgency, and secondary appraisals evaluate the resources available to handle the situation. Historically, this theory aimed to understand individual coping mechanisms in stressful situations. In the health care management literature, it has been frequently used to investigate stress experienced by health care workers.^20^, ^21^ Recently, the focus of appraisal theory has shifted to exploring innovative behaviors, moving away from the stress perspective.^22^, ^23^

In this paper, we argue that moving from primary to secondary appraisal depends on the appraised relevance of the system failure. The threat and challenge response arising from the perceived manageability of the system failure then determines the likelihood of creative behavior as a response.^24^ A maladaptive threat response occurs when there is a perception of insufficient resources. A more positive challenge response, such as SFC, arises when sufficient resources are perceived to be present. At the secondary appraisal stage, the system factors, together with individual self‐efficacy,^25^ determine the behavioral response of health care workers toward system failure.

**FIGURE 1:**
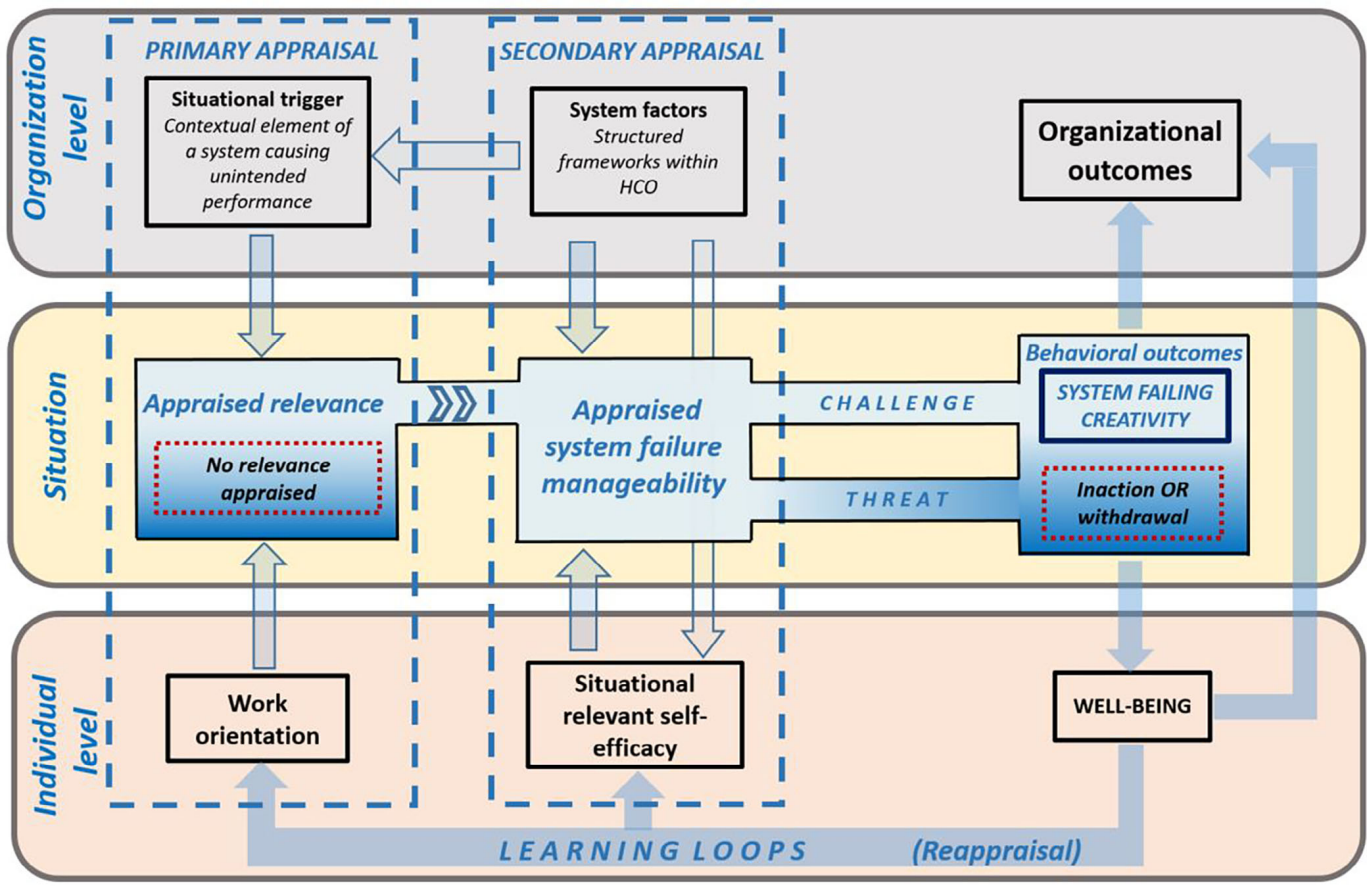
The SFC model.

Following this reasoning, we present a process model for SFC (Figure 1). The remainder of this paper explains the components of the SFC model following the sequence of the SFC process and discusses ways in which HCOs can identify and exploit situations in which health care workers generate creative solutions that can help to optimize patient care.

### 3.1 Appraised relevance

We argue that while a system failure may seem evident, it needs to first be recognized by individual health care workers in the HCO. The recognition of a system failure depends both on situational triggers and the work orientation of the individual. Upon this recognition, the individual appraises the situation to understand the relevance of the situation, which aligns with the *primary appraisal*.

#### 3.1.1 Situational trigger

Situational triggers are events that occur when structured frameworks within an HCO are not working as designed. These structured frameworks are composed of various aspects that together shape the processes within the HCO. Examples include managerial decisions, guidelines and protocols, the layout of physical spaces, organizational hierarchy, and IT infrastructure. Although primarily aimed to ensure patient safety and efficiency,^26^, ^27^ these structured frameworks may become disconnected from clinical practices over time, leading to significant problems.^28^ For instance, a study by Rycroft‐Malone et al.^29^ found that in the cardiac surgical unit, the required care process was designed only for straightforward cases, resulting in inflexibility that couldn't accommodate complex cases, and nurses described the clinical guidelines as overly prescriptive and rigid, even when used as checklists at the end of a procedure.

Another aspect of structured frameworks is hierarchy. Hierarchical structure is often used to manage care practices in HCOs; hierarchy is seen to facilitate leadership flows, streamline responsibility relations, and create a learning setting.^30^ However, mistakes that could potentially harm or be fatal to patients have been shown to occur due to the reluctance of individuals to challenge colleagues in authority when something goes wrong.^31^ Similarly, while information systems improve patient safety and care delivery effectiveness, they too could cause system failures. Lack of interoperability and poor data quality can result in hazardous situations as well.^32^ Although these structured frameworks aim to enhance care quality, previous research indicates they can yield counterproductive effects in certain situations,^31^, ^32^ ranging from minor issues like inefficient protocols to major obstacles such as material access. Both minor issues and major obstacles can serve as situational triggers that initiate the SFC process, depending on the work orientation of the health care worker.

#### 3.1.2 Work orientation

The health care workers' work orientation toward practice improvement consists of extrinsic and intrinsic factors that shape the individual's attitude, beliefs, and values about work.^33^ Extrinsic factors include organizational influences, such as well‐defined goals, leadership support, and culture. These factors serve as extrinsic motivators, which can lead to orientation for quality improvements.^34^ Other extrinsic factors include typical normative pressures in health care. For example, health care providers, especially physicians, may feel pressured to express commitment to the work for altruism, saving lives, and helping communities to uphold the standards of “professionalism” in the industry.^35^ In contrast, intrinsic factors emanate from the deeply personal and profound significance health care holds for many of its workers, driven by the aim to improve health and save lives.^36^ Many view their work as a calling, a description rooted in the philosophical origins of health care as a healing profession.^37^

As shown in Figure 1, the relevance appraisal of a system failure can lead to two outcomes. In one scenario, the individual may acknowledge the occurrence of a system failure but may not perceive a compelling necessity based on their work orientation to take action to address it. Conversely, in another scenario, if the individual appraises the perceived system as significant to their work, influenced by their attitudes, beliefs, and values, they are then likely to appraise the extent to which the system failure is manageable.

### 3.2 Appraised system failure manageability

The system factors and situational relevant self‐efficacy are the SFC model components that determine the system failure manageability appraisal (see Figure 1). We posit that the combination of system factors and self‐efficacy influences the extent to which the worker appraises the situation as manageable in terms of their creative behavior. This perspective aligns well with the dynamic componential model of creativity,^1^ which highlights the impact of the environment on the creative actions of individuals. This section further elucidates the significant role of system factors and situational relevant self‐efficacy, as well as their interaction, on the individual's appraisal of the system failure's manageability.

#### 3.2.1 System factors

Given the inherent complexity of HCOs as complex adaptive systems (CASs),^38^ we adopt the CAS framework to understand the non‐linear, unpredictable behavior of the interdependent parts of the HCO to formulate system factors that influence the system failure manageability. McDaniel Jr. et al.^39^ previously applied the appraisal theory to elucidate health care workers' reactions to “negative surprises” within HCOs as CASs. Combining the CAS framework with appraisal theory increases our understanding of how system factors influence the appraisal of system failure manageability can be achieved.

Formal structured system factors, such as bureaucratization,^40^ centralized or decentralized decision‐making,^41^ and resource constraints,^42^, ^43^ can positively or negatively impact the creativity processes of health care workers. System factors related to the social processes among individual health care workers within an HCO can also impact creativity processes. These include communication,^44^ organizational culture,^45^ and psychological safety,^46^ which can affect the problem‐solving abilities of health care workers and therefore the likelihood of engaging in SFC during the course of routine work.

Furthermore, as indicated by the additional arrows originating from the system factors box in Figure 1, system factors also influence two other aspects of the SFC process. First, they can serve as the situational triggers contributing to the perception of a system failure. It is important to note that the impact of system factors on the relevancy appraisal is distinct from the system failure manageability appraisal. For instance, a protocol may limit the autonomy of a health care worker when performing a routine task (causing a system failure), while the same health care worker may not perceive restrictions on autonomy when attempting to creatively address the same protocol. Second, as shown by the last arrow leading from the system factor box, the distinct set of system factors present during a system failure uniquely affects the self‐efficacy of individual health care workers.

#### 3.2.2 Situational relevant self‐efficacy

We link the concept of self‐efficacy to appraisal theory to underscore how perceived self‐efficacy shapes an individual's behaviors in various situations.^25^ We segment self‐efficacy into three parts: (1) self‐efficacy expectancy, (2) outcome expectancy, and (3) outcome value.^47^, ^48^ These three components can be linked to self‐efficacy and the context of the system failures, enabling the theoretical construction of *situational relevant self‐efficacy*.

*Self‐efficacy expectancy* relates to an individual's belief in their ability to handle specific situations based on their skills and past experiences. For instance, an IT‐skilled nurse might have higher self‐efficacy in addressing an IT‐related system failure than in general administrative tasks. Several organizational aspects can negatively influence self‐efficacy expectancy and thus hinder creativity by instilling the belief that engaging in creative behavior is likely to be unsuccessful. Organizational cultures that discourage creative ideas or enforce strict hierarchies, reducing employee autonomy, are examples of such hindrances.^49^

*Outcome expectancy* is the motivational process where individuals evaluate the potential results of their actions, which significantly impacts creative intentions.^16^, ^50^ For example, a health care worker who expects their creative idea for addressing the system failure will be implemented and effectively resolve the problem will likely demonstrate greater self‐efficacy. Individuals' confidence in expressing creativity is influenced by perceived organizational norms, which can diminish or enhance the belief in generating effective solutions.^51^

*Outcome value* delves deeper into an individual's motivation to address a system failure. While less studied,^48^ the common assumption is that a high outcome value is necessary for self‐efficacy to influence behavior. Health care workers may avoid addressing a system failure if they believe the outcome is not worth the effort or if they perceive that the organizational culture impedes broad adoption of innovative solutions.

The interdependent relation between the HCO's system factors and situational relevant self‐efficacy demonstrates that it is not simply about the presence of certain system factors but rather the degree to which they are present.

### 3.3 Appraisal response: challenge and threat

Lazarus and Folkman^19^ suggested that the outcome of the secondary appraisal elicits emotions, which determine the individual's subsequent behavior. A strong connection can be identified between appraisal theory and creativity given the established theories about the role of emotions on creative behavior in the literature.^52^, ^53^, ^54^ According to Blascovich and Mendes,^24^ the elicited emotions can be categorized in two different responses: a challenge and a threat.

The *challenge response* happens when an individual experiences a positive or negative mood after appraising the system failure manageability that drives the expression of creative behavior. Nijstad et al.^54^ unfold the cognitive mechanisms through which creativity can be achieved through both these types of moods. They argue that positive emotions activate creativity through cognitive flexibility (flexible processing of divergent information, resulting in more novel solutions^53^), whereas negative emotions activate creativity through cognitive persistence (persistent probing and systematically combining elements and possibilities^52^). Hence, both positive and negative moods can drive a challenge–response, each leading to distinct creative outcomes based on the different ways in which they utilize their available resources (i.e., the system factors and their situational relevant self‐efficacy).

In the context of a *threat response*, it is important to note that neither positive nor negative moods always drive creative behavior. Both the positive and negative mood categories include activating, deactivating, and neutral moods concerning creativity.^55^ For example, activating moods are happiness (positive) and anger (negative), while deactivating or neutral moods are relaxed (positive) or sadness (negative). Appraisal theory suggests that such an emotional outcome from assessing a system failure can result in maladaptive behavior.^19^ Given this potential outcome of the SFC process, a health care worker might be inclined to refrain from expressing any creative behavior due to their judgment that they are unable to manage the system failure appropriately.

### 3.4 Behavioral outcomes

The behavioral outcomes of the SFC model illustrate the reaction of a health care worker to the perceived system failure (see Figure 1). We place this reaction, determined by the challenge or threat response of the system failure manageability, in the literature of job (dis)satisfaction with the corresponding behavioral reactions and elaborate on the impact of it on the overall well‐being of the health care worker and the organizational outcomes.

#### 3.4.1 Expressing SFC

There are favorable outcomes associated with the expression of SFC that impact the well‐being of the individual health care worker, organizational outcomes, and the establishment of a positive learning loop for SFC to occur in future system failures. Creative behavior has been found to positively impact both job satisfaction^56^ and individual well‐being.^57^ Additionally, when an organization fosters an environment that embraces creativity among its members—of which SFC is a prime example—this positive influence extends beyond the individual. Specifically, creative behavior displayed by one worker can improve the well‐being of their colleagues.^58^ Moreover, research has demonstrated that an increase in worker well‐being positively affects organizational performance.^59^, ^60^

However, well‐being is not the only factor that contributes to organizational outcomes from the SFC process. An organization that effectively leverages emergent creative ideas can gain significant advantages, including a flow of innovations and an improved problem‐solving capacity.^12^, ^13^ Nevertheless, it is up to the HCO to actively support and embrace emergent valuable ideas and implement them throughout the organization in the appropriate scope. Lastly, the positive outcome resulting from appraisals in the SFC process can impact work orientation as well as self‐efficacy of health care workers. As a result, when facing a subsequent situational trigger, it is more likely for this trigger to be deemed as a relevant system failure that can be creatively addressed. This arises from the recalibration of the individual's work orientation and enhanced self‐efficacy resulting from previous experiences in resolving system failures. These learning loops contribute to the potential for continuous improvement in care over time. In contrast, a negative appraisal can have detrimental effects.

#### 3.4.2 Inaction and withdrawal

A threat appraisal of the manageability of the system failure is likely to result in one of two behavioral outcomes. Organizational research on behavioral outcomes following negative perceptions of work, such as burnout or job dissatisfaction, suggests that employees are inclined to withdraw their effort and commitment, or exit from the workplace.^61^ Health care workers report among the highest levels of burnout and job dissatisfaction of any professional group.^37^ More than half of US physicians, nurses, and administrators report burnout from and dissatisfaction with their jobs.^14^ Job dissatisfaction refers to low contentment with one's job.^62^ It is an emotional state that can precipitate emotional decoupling of a person's sense of self from their work role^62^ and result in outcomes considered counterproductive from an organizational standpoint, such as withdrawal of commitment and effort (i.e., withdrawing effort, or “quiet quitting”), or exit from the workplace.^61^ Considering that the health care sector increasingly suffers from these challenges, a threat response is also possible, especially by dissatisfied people. These behavioral outcomes of a system failure can have a detrimental effect on the well‐being of the individual health care worker.

Besides the decrease in well‐being, a negative appraisal can lead to a decrease in the individual's work orientation, characterized by changes in beliefs, norms, and attitudes that diminish their commitment to the goals of the HCO and their own work. Consequently, their responsiveness to future system failures may decrease, as they are more likely to perceive them as irrelevant. Regarding self‐efficacy, the prior experience of not feeling able to have addressed a previous system failure negatively affects situational relevant self‐efficacy in the subsequent system failure. This can bring a stop to the individual's effort toward continuous improvement over time and result in missed opportunities for the HCO to benefit from valuable creative ideas.

## 4 CONCLUSION

The SFC model describes the process by which creativity can emerge in response to a system failure. Importantly, this form of creativity arises more naturally (i.e., without the deliberate cultivation or facilitation by HCOs, such as through innovation tournaments or dedicated innovation departments) and is characterized as responsive and proactive.^18^ It exemplifies how professionals can exhibit agency by proactively responding to improve the system. The SFC model offers a nuanced view of system failures, illustrating their possible strategic exploitation to achieve positive outcomes, and highlighting the interplay of individual, situational, and organizational system factors. Health care managers and leaders can attune themselves to design and monitor system factors that might lead to system failures and support individuals in these acts of SFC—so they are not seen as covert and interpersonally risky but instead as acts of support for organizational improvement—learning opportunities that can be converted into potential innovation development opportunities. We do not encourage leaders to enforce SFC due to potential safety risks for HCOs. Instead, we recognize that system failures are common in health care and encourage leaders to see their possible benefits. Additionally, understanding how these failures affect health care workers' well‐being can provide valuable insights for leaders. Recognizing the significance of feedback loops, leaders can emphasize the importance of supporting and rewarding creative ideas that emerge from the workforce. By valuing and acknowledging these ideas, leaders can create an environment where future efforts are highly valued.

The SFC model not only provides practical guidance for health care leaders but also offers a framework for researchers to delve deeper into this phenomenon. The SFC model serves as a conceptual model that establishes connections between various concepts based on previous studies highlighting specific relationships. However, comprehensively understanding SFC requires investigating these relationships in a coherent and interdependent manner. Furthermore, while the original appraisal theory considers the influence of coping on regulating elicited emotions, the SFC model does not incorporate this, indicating the need for further exploration of how coping might influence the SFC process. We cannot provide a comprehensive toolkit for investigating SFC yet, but the relationships and interdependencies we have outlined establish a solid foundation for future research in this domain.

## CONFLICT OF INTEREST STATEMENT

The authors declare no conflict of interests.

## Supporting information


**Data S1.** supporting Information.
