# Supplementary material for: System‐failing creativity in health care
Source: Learn Health Syst. 2024 Jun 24;9(1):e10437. doi: 10.1002/lrh2.10437 (PMC11733441; doi:10.1002/lrh2.10437)
Supplement: Supplementary file 1 — Data S1. supporting Information. [file LRH2-9-e10437-s001.docx]

**Appendix A**

**Detailed description of methodological process**

1. ***Collaboration approaches***

In 2021, during the Academy of Management (AOM) Annual Meeting, a session about international collaboration in times of COVID was held focusing specifically on "Maintaining the health care workforce." This session emerged from a collaborative project that one of us worked on (which was recently published, see reference [15] in the paper), who also served as the facilitator. The initiative aimed to support collaborative efforts to scholars, who faced challenges in this regard due to the COVID-19 pandemic, by forming subgroups dedicated to specific issues and potential collaborations. The initial brainstorming session identified crisis leadership and healthcare worker well-being as key areas of interest, with a particular focus on the role of creativity as a potential explanatory factor for workarounds. Participants who were interested in further developing these ideas were asked to express their interest. One week following this session, the collaboration officially commenced with forming a group.

In the subsequent months, we held frequent meetings and gathered a substantial amount of literature. The methods we used for our literature review are detailed in the next section. We used Atlas.ti software to analyze this material, focusing on the various definitions, conceptualizations, and measurements of creativity in healthcare, as well as the research methods used. This analysis led to a discussion on the distinctive characteristics of creativity in healthcare compared to other sectors. Furthermore, we reached out to editors with a proposal for an article that would outline our concept of creativity in this field, receiving positive feedback in response.

After 5 months, we transitioned to a more informal process, using Google Drive to share and discuss sources and manage a ‘living’ collaborative document that everyone could contribute to. Our group meetings were guided by a semi-structured agenda: the progress made between meetings determined the points for discussion for the next meeting. In the last year, once we completed a draft, we adopted a system of sequential feedback rounds and held targeted bilateral meetings between team members from various disciplines. This method enabled us to more effectively merge diverse theories from different scientific fields.

1. ***Literature review approaches***

The literature review process we employed can be described in three phases. Initially, following the workshop, we conducted a scoping review to broadly explore the subject (09/2021-02/2022). This phase involved collecting a diverse range of materials—including scientific articles, news pieces, books, and essays—related to creativity, healthcare, leadership, crisis management, and well-being. This helped develop a common understanding among team members of how these themes intersect. At this stage, without specific inclusion criteria, we gathered about 150 sources. Subsequently, once we identified our goal of creating a process model linking these aspects, we undertook a more targeted explorative review (02/2022-01/2023). This phase utilized the interdisciplinary expertise of our research team to locate theories that could bridge the various themes. Therefore, we focused our search on the combination of aspects we identified. In the following section about our theory selection, we will provide more details on how we identified and chose these theories. The last phase involved a purposeful review aimed at finding case studies and other literature that confirmed or contradicted the relationships and connections identified earlier, leading to the iterative refinement of the model (02/2023-11/2023).

1. ***Selection of theories and model refinement***

Following our scoping review, we identified shortcomings in existing frameworks that failed to adequately explain creativity during system failures. Consequently, we looked for theories that could effectively combine elements of failure, creativity, and well-being. Initially, we aimed for a model depicting these elements as interconnected but without implying directionality or phases. Upon reviewing our initial notes and our collected literature, we concluded that it is the situational impact of a failure that made the form of creativity we were investigating so unique. Furthermore, by viewing creativity as a form of behavioral expression, we established a logical connection to individual well-being based on our targeted exploratory review. This connection was established through psychological theories that explore the relationship between behavior expression and well-being. Having two psychologists on the team provided the necessary expertise to draw these conclusions. Based on these insights, we determined that it was necessary to develop a process model to conceptualize these relationships. We initially drew on the dynamic componential model of creativity and innovation in organizations of Amabile and Pratt, which contextualizes individual creativity within an organizational setting. However, as this model did not fully capture the type of creativity we were after, we gradually moved away from it while still applying its theoretical perspective to link organizational contexts with individual behavior.

After exploring several theories, the one that effectively encapsulated the situational impact of an event on individual behavior was the appraisal theory. This theory formed the basis of our process model. The selection of theories was guided primarily by this decision and required us to define each element as either a starting point, a mediating point, or an endpoint, necessitating clear explanations of the relationships between elements. Each component—such as failure, organizational structures, creative behavior, and well-being—needed to be interconnected in the right order. We chose theories based on their ability to provide robust explanations for these connections. For instance, appraisal theory suggests that an individual's behavior is determined by the elicited emotions resulting from the primary appraisal. Since we defined creativity as the behavioral outcome in our model, it was essential to find a theory that linked emotional states and moods with the expression or non-expression of creative behavior. Initially, we attempted to explain this using various psychological moods, but integrating the dual pathway to creativity theory offered a more compelling explanation. By examining purposefully reviewed empirical literature (02/2022-11/2023), which explored these relationships conceptualized within the SFC model, we were able to confirm or refute them. This evaluation process was used to select the theories for the SFC model, including the Complex Adaptive System (CAS), work orientation, and self-efficacy. This helped solidify the process model based on empirical evidence.
